# Acute *Blastocystis*-Associated Appendicular Peritonitis in a Child, Casablanca, Morocco

**DOI:** 10.3201/eid2101.140544

**Published:** 2015-01

**Authors:** Emilie Fréalle, Dima El Safadi, Amandine Cian, Estelle Aubry, Gabriela Certad, Marwan Osman, Agnès Wacrenier, Emmanuel Dutoit, Colette Creusy, François Dubos, Eric Viscogliosi

**Affiliations:** University of Lille, University Hospital Center, Lille, France (E. Fréalle, E. Aubry, A. Wacrenier, E. Dutoit, F. Dubos);; Pasteur Institute of Lille, Center for Infection and Immunity of Lille, Lille (E. Fréalle D. El Safadi, A. Cian, G. Certad, M. Osman, E. Viscogliosi);; Doctoral School of Sciences and Technology, Lebanese University, Tripoli, Lebanon (D. El Safadi, M. Osman);; Hospital Group of the Catholic Institute of Lille, France (C. Creusy)

**Keywords:** Blastocystis, blastocystosis, parasite, gastrointestinal, appendicitis, peritonitis, Morocco, Casablanca, outbreak, subtypes

## Abstract

Despite increasing reports that *Blastocystis* infection is associated with digestive symptoms, its pathogenicity remains controversial. We report appendicular peritonitis in a 9-year-old girl returning to France from Morocco. Only *Blastocystis* parasites were detected in stools, appendix, peritoneal liquid, and recto-uterine pouch. Simultaneous gastroenteritis in 26 members of the child’s family suggested an outbreak.

*Blastocystis* is a genus of anaerobic protozoan parasites that infect humans and a vast range of animal species. Prevalence in humans varies from 0.5%–24% in industrialized countries to 30%–76% in developing countries (*1*,*2*). Classic clinical features of infection include gastrointestinal symptoms such as nausea, anorexia, flatulence, and acute or chronic diarrhea. Fever is usually absent. An association with irritable bowel syndrome and extraintestinal manifestations such as urticaria has been suggested (*2*). Reports about invasive infection or disseminated diseases are rare (*3*). Here, we report the case of a pediatric patient infected with *Blastocystis* that was manifested by gastroenteritis associated with suppurative appendicitis and peritonitis.

## The Study

In August 2013, a 9-year-old girl who was returning to France after a 1-month stay with her family in Casablanca, Morocco, was admitted to Lille University Hospital in Lille. Symptoms started in Casablanca 3 days before hospital admission and included fever, severe diarrhea (>10 liquid defecations/day), vomiting, and abdominal pain in the hypogastric area and in the right and left lower quadrants associated with bilateral dorsal pain, anorexia, and weakness. 

Blood count showed 13,850/mm^3^ leukocytes (75.4% neutrophils, 15.9% lymphocytes, 8.5% monocytes). C-reactive protein level was increased at 266 mg/L (Low risk: <1.0mg/L; average risk: 1.0–3.0 mg/L; high risk >3.0 mg/L). Traveler’s gastroenteritis was diagnosed, and symptomatic treatment with acetaminophen, phloroglucinol glucoside, and acetorphan was prescribed. However, abdominal pain increased, and total food intolerance occurred in the following hours. 

An abdominal ultrasound was performed, revealing appendicitis with suppuration in the recto-uterine pouch and a reflex ileus. Parasitologic examination of fecal matter revealed only abundant *Blastocystis* vacuolar forms, with >5 parasites per field. We further confirmed absence of *Cryptosporidium* spp. using glycerin assay and real-time PCR. Yeasts and a multimicrobial flora were present in the fecal material, but other infectious agents such as *Salmonella* spp*., Shigella* spp*., Campylobacter* spp.*, Yersinia enterocolitica,* adenovirus, and rotavirus were not detected. Similarly, multimicrobial flora, but no pathogenic bacteria, were detected in the peritoneal liquid and recto-uterine pouch (Table). Histopathologic observation revealed acute suppurative appendicitis with ulcerations extending deep into the muscularis, which was covered with a suppurative and fibrinous exudate. We observed infiltration by numerous neutrophils, eosinophils, plasma cells, and lymphocytes through all layers and into the serous membrane (Figure, panel A). 

**Table:** Microbiological examination of samples of feces, appendix, recto-uterine pouch, and peritoneal fluid from child who had peritonitis, Casablanca, Morocco*

Variable	Date of sampling, 2013						
	Aug 30		Sept 1				Nov 13
Procedure/result	Feces		Appendix	Recto-uterine pouch	Peritoneal fluid		Feces
Microscopic examination	Numerous *Blastocystis *vacuolar forms; no *Cryptosporidium* or other parasites		Presence of rare *Blastocystis*†	ND	ND		Absence of parasites
Real-time PCR							
*Blastocystis* spp.	Positive		Positive	Positive	Positive		Negative
*Cryptosporidium* spp.	Negative		Negative	Negative	Negative		ND
Sequencing							
*Blastocystis* spp. genotype	ST2, ST3		ST3	ST3	ST3		ND
Bacterial culture	Negative for *Salmonella*, *Shigella*, *Campylobacter*, *Yersinia enterocolitica*		ND	Multimicrobial flora	Multimicrobial flora		ND
Viral antigen detection	Negative for aenovirus and rotavirus		ND	ND	ND		ND
*ND, not done; ST, subtype. †Figure, panels B,C.							

**Figure:**
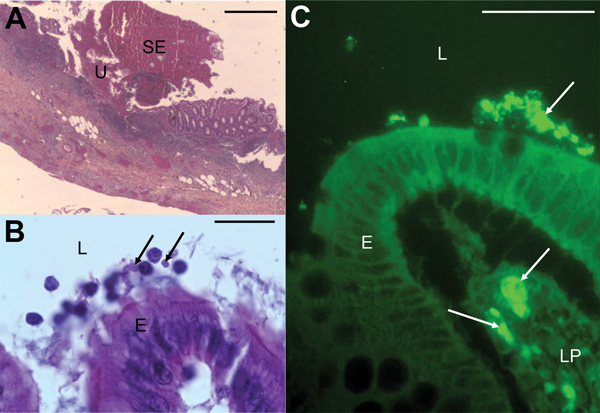
Micrographs showing histopathologic examination of appendix samples from a child who had peritonitis, Casablanca, Morocco, 2013. A) Ulceration (U) covered with suppurative and fibrinous exudates (SE) (hematoxylin-eosin stain). Scale bar indicates 200μm. B) *Blastocystis* parasites (arrows) in the lumen (L), and at the surface of the epithelium (E) (hematoxylin-eosin stain). Scale bar indicates 20μm. C) *Blastocystis* parasites (arrows) in the lumen, at the surface of the epithelium and in the lamina propria (LP) of the mucosa (immunofluorescence labeling with anti-*Blastocystis* ParaFlorB antibody). Scale bar indicates 50μm.

After hematoxylin-eosin staining and immunofluorescence labeling by using the anti-*Blastocystis* Paraflor B monoclonal antibody (Boulder Diagnostics, Marlborough, MA, USA), we detected parasitic forms in the lumen and in the lamina propria of the mucosa (Figure, panels B, C). We used real-time PCR for *Blastocystis* parasite detection as described (*4*), using DNA extracted from stools, appendix, peritoneal liquid, and the recto-uterine pouch, which all tested positive. We subsequently performed small subunit rDNA (SSU rDNA) amplification, then cloned the PCR product and sequenced 5 clones from all the DNA samples to subtype (ST) *Blastocystis* isolates and detect mixed infections (*5*). We identified ST3 in all the analyzed compartments. Mixed infection with ST2 and ST3 was detected only in the stools. The SSU rDNA gene sequences obtained in this study have been deposited in GenBank under accession nos. KJ605630–KJ605649. 

The child completely recovered after an appendectomy, removal of a stercolith from the appendix lumen, and treatment with tinidazole, 20 mg/kg/d, and ceftriaxone, 50 mg/kg/d for 10 days, together with gentamicin, 5 mg/kg/d for 5 days. Although tinidazole is not the first line medication for treatment of *Blastocystis* infection, the child recovered completely and showed total clearance of parasites at day 73: using microscopy and real-time PCR on fecal samples, we found negative results for *Blastocystis*. Data obtained from the child’s mother revealed simultaneous cases of gastroenteritis in 26 family members: 13 adults, 34–98 years of age, and 13 children, 18 months–15 years of age, who lived in the same building at the residential “Mohammadi” area of Casablanca. Adults had mild or moderate diarrhea but symptoms were more severe in children, who all had abundant diarrhea, vomiting, and weight loss. Repatriation in France of an 18-month-old baby was considered, but his condition improved. None of the family members required hospitalization. Unfortunately, no explorations were performed, therfore the diagnosis could not be microbiologically documented.

## Conclusions

Reports of *Blastocystis* infection associated with diarrhea and clinical symptoms in immunocompetent and immunocompromised patients have increased during the past 2 decades (*2*). Tissue invasion of *Blastocystis* parasites in the appendix (*6*) or in the colon mucosa (*3*), associated with acute or chronic inflammation, has been reported. However, controversy still exists over whether this parasite is commensal or pathogenic; this case further supports its invasive and inflammatory potential. Previous reports regarding the presence of *Blastocystis* parasites in 4 of 100 appendix specimens from patients with acute appendicitis (*7*), and of pseudoappendicular illness, which led to appendectomies in children with intestinal infection caused by this parasite (*8*), suggest that *Blastocystis* infection could be associated with appendicitis. Nevertheless, the actual role of *Blastocystis* in the pathogenesis of appendicitis remains inconclusive. In this report, the presence of a stercolith, which can be found in 50%–80% of appendicitis cases, suggested mechanical obstruction of the appendix’s lumen, which is the main etiology of appendicitis. 

Here, we report dissemination of *Blastocystis* into the lumen, the mucosa, and the recto-uterine pouch exudate, associated with appendicular acute inflammation, and no other infectious agent was detected. These observations, together with the well-documented acute or chronic inflammation occurring in humans or animals with *Blastocystis* infections (*3*,*9*), likely support the contribution of this infection to the inflammatory process. Infection with ST3 further reinforced this hypothesis. Indeed, the presence of pathogenic strains among ST3 has been confirmed through experimental infections in rats (*9*). Additionally, a substantial inflammatory reaction and an increased propagation of human colorectal cancer cells exposed to *Blastocystis* ST3 antigens has been demonstrated in vitro (*10*). For humans, the pathogenicity of different STs is unclear and remains a debatable issue. ST1 isolates were found to be more prevalent among symptomatic patients in Lebanon (*5*), but ST3 was found to be the only ST that showed pathogenic potential in Malaysian patients when compared with ST1 and ST2 (*11*). ST3 was also found to be significantly associated with diarrhea in Libya (p = 0.008) (*12*). For this case, the fact that only ST3 was detected in all analyzed samples, whereas a mixed infection with ST2 and ST3 was found in the child’s stools, further supports the high invasive potential of ST3. ST3 is the most common ST in Europe, but, in African countries, its frequency varies from 17.8% in Libya (*12*) to 61.9% in Egypt (*13*). In Morocco, a 28.7% prevalence of blastocystosis has been reported, but data concerning the ST distribution of the parasite are not available (*14*). Furthermore, although *Blastocystis* infection could not be confirmed among the child’s relatives, the simultaneous occurrence of gastroenteritis cases in the same family and the absence of other infectious agents in the child’s stools suggest a potential outbreak of *Blastocystis* infection. *Blastocystis* parasites could have spread within the child’s family, as previously reported in Italy, where 2 adopted children originating from India and the Côte d’Ivoire transmitted *Blastocystis* parasites to their adoptive parents and grandmother (*15*). Possible acquisition of this parasite from a common source such as contaminated water could also explain family transmission in this report. Altogether, these data highlight 1) the need for both systematic parasitologic examinations of stools in patients with invasive infections who are traveling from countries with high *Blastocystis* prevalence and 2) the need for routine provision of imidazoles for empiric treatment of peritonitis.
